# Advances in the mechanism of small extracellular vesicles promoting the development of hepatocellular carcinoma through multi-network fusion

**DOI:** 10.3389/fimmu.2025.1558468

**Published:** 2025-07-09

**Authors:** Xiaoying Yuan, Defa Huang, Liang Peng, Yilong Lin, Lijuan Wang, Jiawei Yan, Youming Qiu, Chenggui Song, Qi Wang

**Affiliations:** ^1^ The First School of Clinical Medicine, Gannan Medical University, Ganzhou, China; ^2^ Department of Laboratory Medicine, The First Affiliated Hospital of Gannan Medical University, Ganzhou, China; ^3^ Department of Research and Education, The Second People’s Hospital of Jingdezhen, Jingdezhen, China; ^4^ School of Medical Technology, Gannan Medical University, Ganzhou, China

**Keywords:** hepatocellular carcinoma, small extracellular vesicles, multi-network fusion, mechanism, therapy

## Abstract

Hepatocellular carcinoma (HCC) is a highly malignant epithelial tumor characterized by global high incidence and poor clinical prognosis. Radical surgical resection, as the standard treatment for early-stage HCC patients, has been extensively validated for its therapeutic efficacy. However, epidemiological studies indicate that most patients are already in advanced stages at initial diagnosis, losing eligibility for radical treatment. Notably, HCC pathogenesis exhibits marked etiological heterogeneity, posing significant challenges for clinical management. Although significant breakthroughs have been made in understanding HCC drivers at pathophysiological levels, translational applications of these findings remain hindered by multiple barriers. Currently, elucidating the molecular mechanisms of HCC pathogenesis and identifying effective therapeutic targets constitute major research priorities in this field.Small extracellular vesicles (sEVs) are phospholipid bilayer vesicles (30-150 nm in diameter) carrying functional proteomes and nucleic acids (e.g., miRNAs, lncRNAs) with substantial biological activity. Studies demonstrate that sEVs contribute to malignant phenotype acquisition by modulating key signaling pathways such as PI3K/AKT and Wnt/β-catenin. These molecular cascades ultimately confer hallmark pathological features including aberrant proliferation, apoptosis resistance, and immune evasion to tumor cells. Within multi-network regulatory systems, sEVs serve as crucial intercellular messengers mediating tumor cell interactions with other tumor microenvironment (TME) components (e.g., cancer-associated fibroblasts, immune cells). Such communication facilitates TME reprogramming, pro-angiogenic phenotypic shifts, and therapy resistance development. Nevertheless, the precise molecular mechanisms of sEVs in HCC pathogenesis remain incompletely understood, warranting further exploration of their translational potential in clinical practice.

## 1 Introduction

Hepatocellular carcinoma (HCC) ranks among the most prevalent and lethal malignancies worldwide, with escalating incidence and mortality rates (1). HCC exhibits multifactorial etiology, with primary risk factors encompassing chronic HBV/HCV infections, alcoholic liver disease, and non-alcoholic fatty liver disease (2). Current therapeutic strategies for HCC merely extend nominal survival curves while inducing broad-spectrum toxicities. This ultimately leads to treatment resistance development in patients (3). Consequently, developing novel therapeutic approaches is imperative. Recent advances in fundamental medical research have progressively unraveled HCC pathogenesis mechanisms. Small extracellular vesicles (sEVs), as critical tumor microenvironment components, have garnered substantial research attention.

sEVs are nanoscale membranous vesicles secreted by diverse cell types, transporting bioactive cargo (proteins, lipids, mRNAs, miRNAs) to mediate intercellular communication and signaling (4). Studies demonstrate HCC-derived sEVs interact not only with tumor cells but also with TME components (fibroblasts, endothelial cells, immune cells), promoting hepatocarcinogenesis and progression via multinetwork fusion mechanisms (5). Although preliminary understanding of sEVs’ mechanistic roles in HCC exists, their complex signaling networks and clinical potential require further exploration.

This review systematically elucidates the multinetwork regulatory mechanisms of sEVs in HCC pathogenesis. Integrating current evidence, we analyze how sEVs drive HCC progression by: (a) modulating pivotal pathways (PI3K/AKT, Wnt/β-catenin); (b) reprogramming TME cellular composition/functionality; (c) enhancing malignant behaviors (proliferation, metastasis). Building upon these mechanisms, we evaluate sEVs’ translational value as precision medicine targets. This review addresses three key questions: (a) sEVs biogenesis/molecular signatures; (b) pathological mechanisms of sEVs-mediated network crosstalk; (c) clinical applications and translational prospects.

## 2 sEVs

### 2.1 Definition and classification of sEVs

The International Society for Extracellular Vesicles (ISEVS) defines extracellular vesicles as phospholipid bilayer-enclosed membranous structures ranging from 30–5000 nm in diameter. Their fundamental biological characteristics include cellular origin, lack of replicative capacity, and intercellular communication functions (6). Current classification criteria are based on physical properties and biogenesis pathways: by size as small EVs (sEVs, <200 nm) and large EVs (lEVs, >200 nm); by origin as exosomes (endosomal pathway), microvesicles (plasma membrane budding), and apoptotic bodies (programmed cell death products). Notably, ISEVS recommends using the operational term “small extracellular vesicles” (sEVs) rather than the mechanistically suggestive “exosomes”. This recommendation stems from: technical limitations in distinguishing biogenesis pathways; absence of specific molecular markers; and substantial heterogeneity in clinical samples (6, 7).

### 2.2 Molecular characteristics and characterization techniques of sEVs

sEVs exhibit characteristic nanoscale size distribution (30–200 nm) and marked morphological heterogeneity. Their bilayer membranes are enriched with tetraspanins (CD63/CD81/CD9) and tissue-specific markers (8, 9). Modern characterization techniques include: (a) Nanoparticle tracking analysis (NTA) for size quantification; (b) Transmission electron microscopy (TEM) for ultrastructure; (c) Super-resolution microscopy overcoming optical limits; (d) Mass spectrometry for molecular profiling (10, 11). Key technical challenges persist: *in vitro* models are culture-condition dependent (e.g., FBS starvation alters proteomes) (12); xenografts fail to recapitulate full TME interactions (13); clinical samples suffer lipoprotein co-isolation (plasma concentration ~10^16^/ml) (14). Optimization strategies combine separation techniques (e.g., SEC-density gradients) and surface marker capture, requiring purity-yield tradeoffs (15).

### 2.3 Biogenesis and uptake of sEVs

Rab GTPases are small GTPases belonging to the Ras superfamily that primarily regulate intracellular membrane trafficking and vesicular transport (16). They cycle between GTP-bound (active) and GDP-bound (inactive) states to modulate functional status, recruiting effector proteins to specific membrane compartments to control vesicle formation, trafficking, and fusion (17). This mechanism is crucial for the biogenesis of small extracellular vesicles (sEVs).

sEVs formation initiates with membrane invagination of early endosomes to generate intraluminal vesicles (ILVs), which subsequently develop into multivesicular bodies (MVBs) (18). Rab GTPases influence sEVs production and release by regulating multiple steps of this process. For instance, Rab27a and Rab27b promote MVB docking with the plasma membrane to enhance sEVs secretion (19), while Rab7 determines whether MVBs undergo degradation or sEVs release (20). Furthermore, Rab11- and Rab35-regulated sEVs secretion appears ESCRT-independent but Rab27-dependent for ILV formation (21), demonstrating the diverse functions of Rab proteins in sEVs biogenesis. Distinct Rab proteins precisely control sEVs generation through specific effector protein networks. Rab5 initiates ILV formation at the early endosome stage (22), while Rab11 affects the recycling endosome pathway (23), collectively ensuring proper sEVs assembly and function.

**Figure 1:**
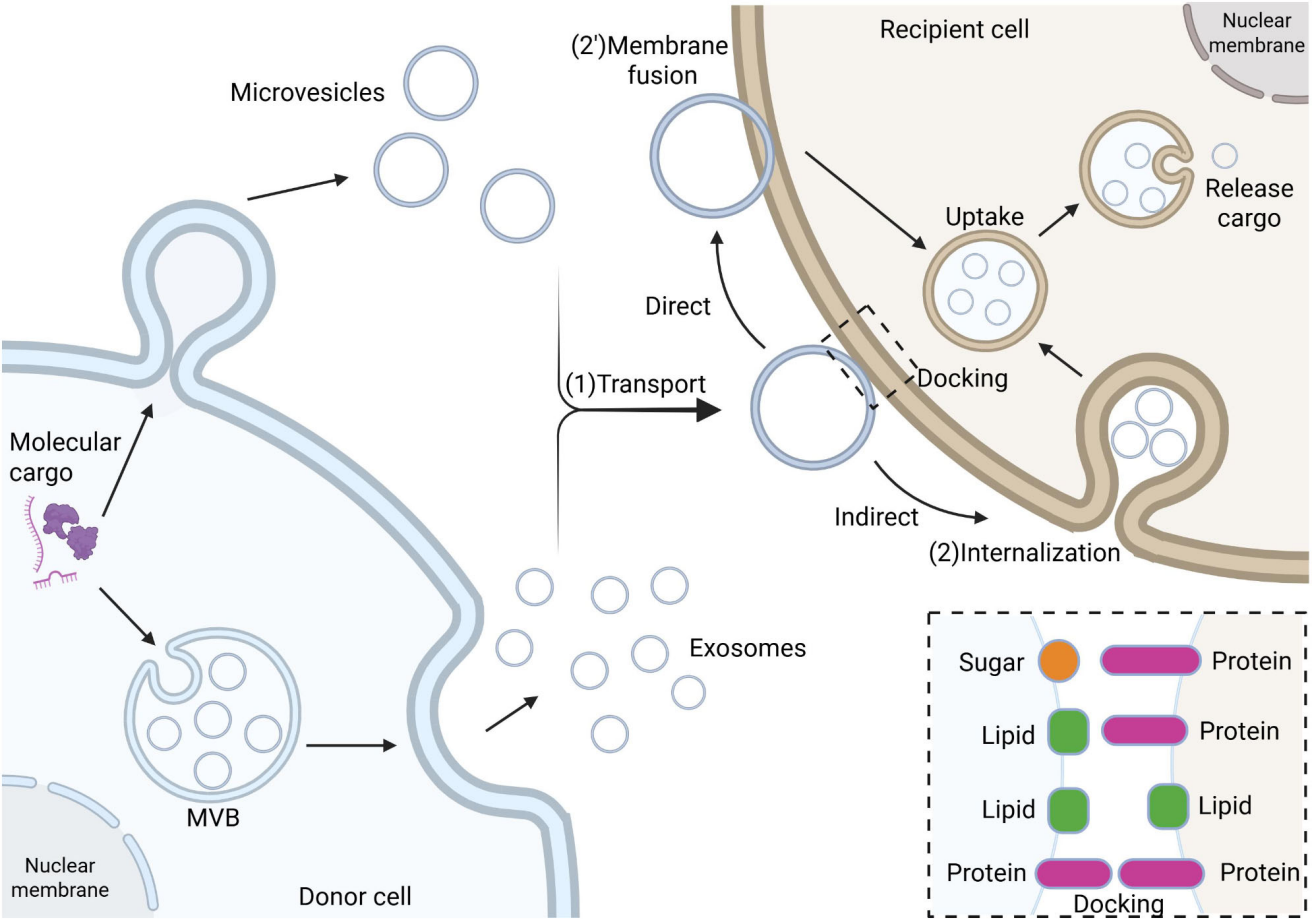
The biogenesis, transport, and internalization mechanisms of sEVs. Biogenesis of sEVs initiates from early endosomes, where the endosomal membrane invaginates to form intraluminal vesicles (ILVs) through membrane budding and sorting mechanisms, which subsequently mature into late endosomes, also known as multivesicular bodies (MVBs). MVBs fuse with the plasma membrane to release their intraluminal sEVs into the extracellular matrix (ECM). During intercellular communication, sEVs bearing surface ligands (e.g., transmembrane proteins or lipids) bind to specific receptors on target cell membranes, followed by internalization via endocytosis (including clathrin-dependent or -independent pathways) or membrane fusion. The newly formed early endosomes undergo maturation in the cytoplasm, ultimately releasing their bioactive cargo (e.g., nucleic acids, proteins) into the target cell cytoplasm, thereby modulating cellular physiological or pathological processes.

Selective uptake of sEVs represents a core aspect of intercellular communication, being tightly regulated rather than stochastic. The membrane protein composition of sEVs serves as a key determinant for selective uptake. Integrin family proteins direct sEVs homing to specific tissues (24), explaining why tumor-derived sEVs preferentially target particular organs. Tetraspanins (CD9, CD63, CD81) mediate cell-specific recognition through interactions with receptor cell surface ligands (25). Multiple mechanisms exist for sEVs entry into recipient cells, including clathrin-dependent endocytosis, caveolin-mediated endocytosis, macropinocytosis, and direct membrane fusion (26) (**Figure 1**).

### 2.4 Research limitations of sEVs

Despite established definitions, high-purity sEVs isolation remains challenging due to incomplete biological understanding. *In vitro* models: Cell line-derived sEVs are experimentally controllable but their biogenesis is altered by artificial conditions (e.g., serum-free media), modifying molecular composition. FBS starvation alters sEVs yield, proteome, protein metabolic regulation, and membrane raft assembly functions (12). Given these effects, sEVs-depleted serum is recommended for *in vitro* studies. Xenograft models preserve tumor characteristics but fail to replicate dynamic TME interactions during tumor growth. Immunodeficient mice lack complete immune environments and human-mouse cellular interactions, limiting translational studies of sEVs-mediated immunomodulation (13). Clinical samples: Plasma-derived sEVs are clinically relevant but show inter-individual variability and lipoprotein contamination, requiring stringent characterization. Current methods (dUC, ExoQuick) co-isolate plasma proteins/lipoproteins and may induce vesicle aggregation. Notably, plasma lipoproteins (~10^16^/ml) share size/density characteristics with sEVs (chylomicrons/VLDL/HDL) (14).

### 2.5 Challenges and optimization in sEVs isolation technology

#### 2.5.1 Inefficient separation of non-vesicular contaminants

Although traditional methods such as ultracentrifugation effectively enrich sEVs, they also co-precipitate contaminants including lipoproteins and protein aggregates (27). The MISEVS2023 guidelines recommend a multi-parametric evaluation strategy, incorporating immunoblotting or mass spectrometry to detect negative markers such as apolipoproteins. Optimization strategies include combining ultracentrifugation with size-exclusion chromatography, employing density gradient centrifugation for enhanced resolution, and exploring emerging technologies such as microfluidics (6). Standardized documentation of isolation methods and contaminant profiles is crucial to ensure reproducibility.

#### 2.5.2 Significant variability in protein marker expression

Commonly used markers such as CD63 and CD9 exhibit heterogeneous distribution across sEVs subpopulations, with expression dynamically influenced by cellular origin and disease state (28). Researchers should employ a combination of universal and cell-specific markers for validation and enhance detection accuracy using advanced techniques such as high-resolution flow cytometry.

#### 2.5.3 Technical bottlenecks in clinical-scale applications

Size-exclusion chromatography suffers from low recovery rates (30-60%) and limited throughput (29), whereas microfluidic technology demonstrates significant advantages, achieving >80% recovery, reducing processing time to minutes, and enabling specific capture of disease-associated sEVs subpopulations (30). Future efforts should focus on standardization through multicenter validation and the development of integrated automated workstations to address scalability challenges.

### 2.6 Single-vesicle analysis technologies

Conventional bulk analysis methods fail to resolve the high heterogeneity of sEVs, driving the need for single-vesicle detection technologies. Next-generation single-vesicle analysis enables precise characterization of individual vesicles’ physical properties and molecular composition, offering novel insights into sEVs biological functions (31).

Advanced microscopy techniques are revolutionizing sEVs observation. Super-resolution microscopy (STORM/PALM) overcomes the optical diffraction limit, revealing nanoscale structural features of sEVs (32). Cryo-EM preserves native sample states, providing authentic 3D morphological information of sEVs (33).

Single-molecule detection significantly enhances sEVs analysis precision. Single-molecule fluorescence tracks dynamic surface interactions, while nanopore sequencing enables direct RNA detection without amplification (34–36). These approaches offer unique advantages for low-abundance biomarker discovery.

Microfluidic platforms provide high-throughput solutions for sEVs analysis. Integrated with fluorescent labeling or Raman spectroscopy, these chip systems enable rapid sorting and characterization of individual sEVs (37). Digital microfluidics advances further by permitting multiplexed analysis of captured single vesicles.

Machine learning algorithms are transforming sEVs data processing. Deep learning models automatically identify characteristic patterns of sEVs subpopulations, while clustering analysis aids in discovering novel functional classifications (38). These methods are particularly suited for handling massive single-vesicle datasets.

Despite promising prospects, single-vesicle analysis faces sEVseral technical challenges. Key issues requiring resolution include balancing sensitivity with throughput, standardizing detection methods, and ensuring clinical translation feasibility. Overcoming these challenges will determine the technology’s practical utility.

Next-generation technologies will focus on multidimensional integrated analysis. Integrating nanotechnology, biosensing, and advanced computational methods, future single-vesicle analysis may achieve higher-precision multi-omics detection. This will open new possibilities for precision medicine and fundamental research.

## 3 Network regulation of tumor microenvironment by sEVs in hepatocellular carcinoma

**Figure 2:**
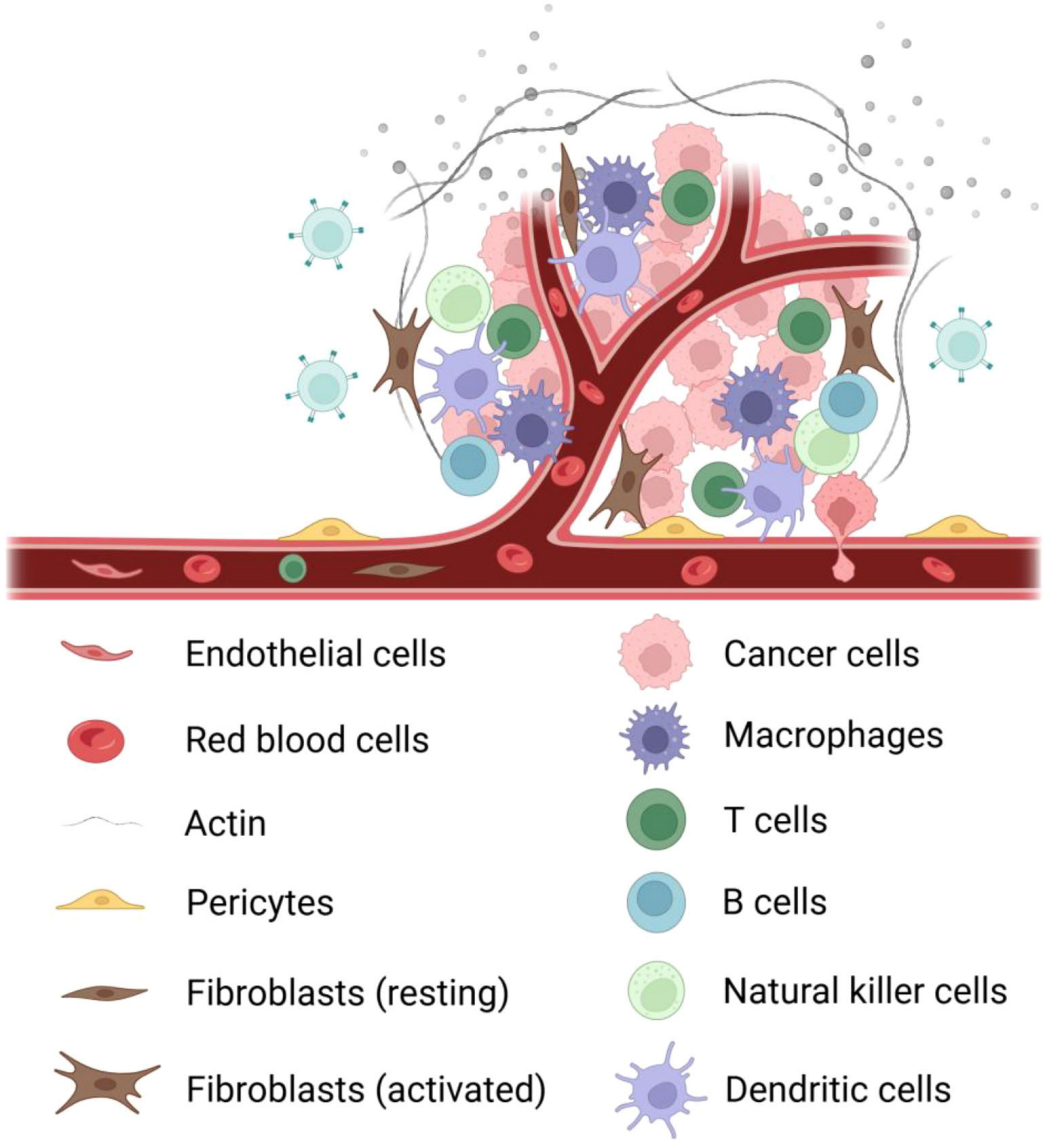
Components of the tumor microenvironment. It mainly contains the following cells: ① tumor cells; ② immune cells: tumor-associated macrophages, T cells, B cells, natural killer cells, dendritic cells; ③ fibroblasts: activated fibroblasts, resting fibroblasts; ④ blood vessels: erythrocytes, pericytes, endothelial cells ; ⑤ extracellular matrix; ⑥ signaling molecules.

The tumor microenvironment (TME) constitutes a complex ecosystem comprising diverse cell types and their secretory factors (39). This system primarily consists of: (a) Tumor cells - the central component exhibiting uncontrolled proliferative potential and invasiveness (40); (b) Cancer-associated fibroblasts (CAFs) - secreting bioactive factors (growth factors, cytokines, ECM components) to critically regulate tumor progression and metastasis (41); (c) Endothelial and pericytes - forming structural/functional units of tumor vasculature that enhance hematogenous metastasis via angiogenesis (42); (d) Immune cells (T/B cells, TAMs, DCs, MDSCs) - collectively participating in immunesurveillance, immunosuppression and immune evasion (43) (**Figure 2**).

sEVs serve as crucial signaling mediators in TME, orchestrating intercellular communication, metabolic reprogramming and immunomodulation (44, 45). By transporting diverse bioactive molecules, sEVs mediate complex crosstalk among tumor, stromal and immune cells to drive malignant progression (46, 47). Metabolically, sEVs remodel TME metabolism by transferring metabolites and regulators to fuel tumor proliferation (48). Notably, sEVs exhibit dual immunoregulatory roles: suppressing effector immune cells while activating immunosuppressive populations to establish an immune-tolerant niche (49). These findings establish sEVs as both essential TME components and promising therapeutic targets, offering novel avenues for treatment optimization and prognostic evaluation.

### 3.1 sEVs in communication network regulation

#### 3.1.1 sEVs and common oncogenic mechanisms in HCC

sEVs participate in the common pathological processes of HCC by mediating intercellular communication. At the molecular regulatory level, the long non-coding RNA HULC competitively inhibits miR-372-3p expression, leading to upregulation of Rab11a protein, thereby promoting sEVs secretion and accelerating HCC progression. Notably, the expression level of HULC in serum sEVs of HCC patients is significantly higher than in healthy controls, suggesting its potential as a diagnostic biomarker (50). In drug resistance regulation, upregulated Rab27B expression in drug-resistant HCC cells enhances sEVs secretion, promoting the efflux of chemotherapeutic agents (e.g., 5-fluorouracil) and reducing intracellular drug concentration; genetic knockout of Rab27B reverses this resistant phenotype (51). Furthermore, sEVs-mediated transfer of circPAK1 is a key mechanism of acquired resistance in HCC, as resistant cells transmit circPAK1 to sensitive cells via sEVs, conferring drug resistance (52).

sEVs facilitate malignant behaviors in HCC by transferring specific RNAs and proteins (53). Upregulation of NEAT1 reduces tumor-suppressive miRNAs (e.g., miR-634, miR-638) in sEVs, enhancing the proliferation and invasion of HCC cells (54). Moreover, sEVs secreted by highly metastatic HCC cells carry carboxypeptidase E (CPE), which can be taken up by low-metastatic cells, promoting their malignant transformation, whereas CPE inhibition reverses this effect (55). Overexpression of p62 protein increases sEVs secretion, enhancing the migration and invasion of recipient cells (56). Ribosomal protein L9 (RPL9) transmits miR-24-3p and miR-185-5p via sEVs, further promoting HCC progression (57). DEAD-box helicase 55 (DDX55) is enriched in HCC-derived sEVs and promotes tumor invasion and angiogenesis through intercellular transfer (58). These findings indicate that sEVs drive the malignant phenotype of HCC by regulating key molecular networks, highlighting their importance in targeted therapy.

#### 3.1.2 sEVs and unique oncogenic mechanisms in HCC

In virus-associated HCC, sEVs exhibit distinct regulatory features. CD81-positive sEVs mediate viral immune evasion and promote tumor progression in HCV-associated HCC; HCV viral particles exploit CD81-positive sEVs for transmission, and this sEVs subpopulation is significantly enriched in HCV-positive HCC patients, suggesting its potential as a therapeutic target (59). In HBV-associated HCC, hepatitis B virus core antigen (HBcAg) delivers miR-135a-5p via sEVs, suppressing VAMP2 expression, thereby enhancing anti-apoptotic capacity and fostering drug resistance in HCC cells (60). Additionally, dysregulated autophagy in HCC patients leads to aberrant release of Glypican-3 (GPC3) in sEVs, and its high expression profile makes it a candidate molecular marker for early diagnosis (61).

#### 3.1.3 sEVs and universal tumor-suppressive mechanisms in HCC

sEVs suppress HCC malignant progression by delivering tumor-suppressive molecules or regulating key signaling pathways. Studies demonstrate that tumor-suppressive long non-coding RNAs (e.g., SENP3-EIF4A1) delivered by sEVs significantly inhibit HCC cell proliferation and induce apoptosis (62). Hesperidin modulates sEVs molecular composition by reducing oncogenic RNA cargo (e.g., RAB11A mRNA and lncRNA-RP11-583F2.2) while upregulating tumor-suppressive miR-1298 expression, thereby effectively inhibiting hepatic precancerous lesion development (63). Resveratrol downregulates Rab27a to reduce sEVs secretion and alters lncRNA SNHG29 expression in sEVs, consequently inhibiting Wnt/β-catenin signaling and autophagy processes (64). The transcription factor KLF4 suppresses HCC progression by upregulating sEVs surface markers CD9 and CD81, whereas low expression of RNA helicase DDX3 promotes sEVs secretion and enhances stemness features and drug resistance in HCC cells (65, 66).

In therapeutic applications, engineered sEVs demonstrate remarkable targeted delivery potential. For instance, anti-GPC3 antibody-modified sEVs efficiently deliver miR-26a, significantly suppressing HCC growth (67). GalNAc-modified sEVs co-deliver paclitaxel (PTX) and miR122, synergistically enhancing antitumor effects (68). Furthermore, sEVs-based gene editing systems show promising applications; AAV6 vectors effectively deliver suicide genes (e.g., inducible caspase 9), markedly enhancing HCC cell killing (69). The CRISPR-Cas9 ribonucleoprotein system delivered by sEVs also exhibits high-efficiency gene editing capability (70). Advanced studies reveal that multiplex siRNA delivery systems (targeting GPX4 and DHODH) enhance sorafenib-induced ferroptosis to overcome HCC drug resistance (71).

#### 3.1.4 sEVs and HCC-specific tumor-suppressive mechanisms

Certain tumor-suppressive mechanisms exhibit HCC-specific regulatory characteristics. Serum cathelicidin antimicrobial peptide (CAMP) levels are significantly reduced in HCC patients, and CAMP supplementation effectively inhibits HCC cell proliferation, suggesting its potential as a diagnostic biomarker (72). Natural killer (NK) cell-derived sEVs selectively target HCC cells and induce apoptosis (73). Additionally, HEK293 cell-derived sEVs delivering miR-365a-3p significantly suppress HCC proliferation and promote apoptosis (74). Notably, Parkinson’s disease cell-derived sEVs enriched with α-synuclein inhibit HCC growth and migration (75).

In summary, sEVs play pivotal roles in HCC pathogenesis, drug resistance development, and metastasis by participating in complex molecular network regulation. These mechanisms encompass both universal HCC regulatory pathways and virus-specific modes of action, highlighting their translational value as diagnostic biomarkers and targeted therapeutic vehicles (**Table 1**).

**Table 1:** Main mechanisms involved in the regulation of communication networks by sEVs.

Effect	Key signals	Main mechanisms	Ref.
Pro-cancer	lncRNA HULC	Inhibition of miR-372-3p expression, up-regulation of Rab11a protein expression, and enhancement of sEVs secretion	(50)
	HCV	Evades immune surveillance by binding to CD81+ sEVs	(59)
	GPC3	Secretion through sEVs as an early diagnostic marker for HCC	(61)
	Rab27B	Exclusion of chemotherapeutic drugs from cells via sEVs reduces intracellular drug concentrations and enhances drug resistance	(51)
	HBc	Up-regulation of miR-135a-5p expression in sEVs, inhibition of its target gene VAMP2, and enhancement of anti-apoptotic and chemotherapy resistance	(60)
	CircPAK1	Delivery to sensitive cells via sEVs, conferring cellular drug resistance	(52)
	NEAT1	Promotes secretion of sEVs and regulates significant down-regulation of oncogenic miRNA expression in sEVs	(54)
	CPE	Low-metastatic cells significantly promote their malignant behavior after uptake of carboxypeptidase E released by high-metastatic tumor cells via sEVs	(55)
	P62	Increased secretion of sEVs, enhanced malignant behavior of receptor cells	(56)
	RPL9	Delivery of specific miRNAs via sEVs	(57)
	DDX55	Enhancement of tumor cell invasiveness and angiogenesis through sEVs delivery between tumor cells and endothelial cells	(58)
	Gremlin-1	Enhancement of invasiveness and metastasis of HCC cells by sEVs, activation of Wnt/β-catenin and BMP signaling pathways, and enhancement of drug resistance	(135)
Anti-cancer	Hesperidin	Significantly decreased the expression of RAB11A mRNA and lncRNA-RP11-583F2.2 in sEVs and increased the expression of miR-1298 in sEVs	(63)
	AAV6	Delivery of an inducible caspase 9 suicide gene and significant enhancement of tumor cell killing	(69)
	lncRNA SENP3-EIF4A1	Delivery of SENP3-EIF4A1 to HCC cells via sEVs. Inhibits their proliferation and migration and promotes their apoptosis	(62)
	KLF4	Inhibition of HCC progression by altering sEVs subtypes through upregulation of sEVs surface proteins CD9 and CD81	(65)
	DDX3	Promoting the secretion of sEVs and enhancing the expression of sEVs-related proteins, thereby promoting stem cell properties and drug resistance in HCC cells	(66)
	miR-26a	Delivery of miR-26a to HCC cells via sEVs significantly inhibits tumor growth	(67)
	siRNA	Delivery of siRNAs targeting GPX4 and DHODH via sEVs significantly enhances the iron death effect of sorafenib in therapy	(71)
	CAMP	CAMP supplementation inhibits the proliferation of HCC cells	(72)
	α-synuclein	Significant inhibition of HCC growth and migration through sEVs uptake by HCC cells	(75)
	Resveratrol	Downregulation of Rab27a expression, inhibition of sEVs secretion, alteration of lncRNA expression in sEVs, inhibition of Wnt/β-catenin signaling pathway and autophagy	(64)
	Hsa-mir-365a-3p	Delivery of hsa-miR-365a-3p to HCC cells via sEVs significantly inhibited cell proliferation, increased oxidative stress, and induced apoptosis	(74)

### 3.2 sEVs in metabolic network regulation

#### 3.2.1 sEVs and hypoxia

The mechanistic role of hypoxic microenvironment in HCC progression has been extensively elucidated. Hypoxia regulates HCC malignancy through sEVs-mediated mechanisms. Hypoxic conditions modulate sEVs secretion via HIF-1α, influencing HCC proliferation, metastasis, and immune evasion. HIF-1α facilitates GPC3 loading into sEVs, reducing intracellular GPC3 to suppress Wnt/β-catenin signaling and tumor growth (76). Hypoxia-regulated sEVs biogenesis promotes angiogenesis through miRNA transfer. HIF-1α upregulates miR-3174 under hypoxia and enhances its packaging into sEVs. These sEVs are delivered to endothelial cells, inhibiting HIPK3 signaling to enhance angiogenesis/vascular permeability and accelerate HCC metastasis (77).

Regarding sEVs-mediated malignant transformation, miR-1273f activates Wnt signaling to promote HCC invasion. Hypoxic HCC-derived sEVs enriched with miR-1273f activate Wnt/β-catenin signaling to enhance proliferation, migration, and EMT (78). Further studies reveal hypoxic sEVs alter hepatocyte mechanical properties. Hypoxic sEVs (H-exos) promote proliferation/migration at lower concentrations than normoxic sEVs, inducing cytoskeletal reorganization and reduced elastic modulus (79). Crucially, hypoxic sEVs induce malignant transformation of normal hepatocytes. Chronic hypoxia enables HCC-sEVs to transform HL-7702 cells, enhancing proliferation/migration, tumor marker expression, and mechanical changes, while promoting tumor growth and liver damage *in vivo (* 80).

Regarding metastasis, sEVs facilitate pre-metastatic niche formation. Hypoxic HCC-sEVs activate fibroblast ERK1/2-NFκB signaling to establish pulmonary PMN. Oleanolic acid (OA) inhibits this pathway to block PMN formation, showing anti-metastatic potential (81). Recent studies show CAF-derived sEVs containing circHIF1A promote immune evasion. Hypoxic CAF-sEVs deliver circHIF1A to stabilize PD-L1, enhance malignancy, and suppress CD8+ T cells, suggesting immunotherapeutic targets (82).

#### 3.2.2 sEVs and glycolysis

HCC-derived sEVs regulate glycolysis via lncRNA transfer to promote progression. sEVs-carried ZFPM2-AS1 suppresses miR-18b-5p to upregulate PKM, activating HIF-1α-dependent glycolysis and enhancing HCC malignancy. ZFPM2-AS1 also promotes M2 macrophage polarization to accelerate progression (83). sEVs-delivered miR4458HG binds IGF2BP2 to stabilize HK2/SLC2A1 mRNAs, enhancing glycolysis and HCC growth (84).

circRNAs modulate HCC glycolysis via miRNA sponging. circFBLIM1 (enriched in HCC-sEVs) sequesters miR-338 to derepress LRP6, promoting glycolysis (85). Similarly, circ-ZNF652 inhibits miR-29a-3p to upregulate GUCD1, enhancing glycolytic flux - its knockout suppresses HCC glycolysis (86).

Highly metastatic HCC cells (e.g., 97H/LM3) secrete sEVs enriched with glycolytic/gluconeogenic/PPP proteins to enhance invasiveness (87). Conversely, senescent HCC cells deliver miR-146a-5p via sEVs to suppress glycolysis. This miRNA targets IRF7 to downregulate PFKL, reducing glucose metabolism and tumor growth (88).

#### 3.2.3 sEVs and other metabolic pathways

FTO demethylates GPNMB mRNA to stabilize its expression and promote sEVs loading. sEVs-delivered GPNMB binds SDC4 on CD8+ T cells to suppress activation, enabling immune evasion. This FTO/m6A/GPNMB axis reveals key HCC mechanisms and therapeutic targets (89).

HCC cells enhance sEVs biogenesis/secretion via ferroptosis to clear misfolded proteins and alleviate ERS. Unsaturated fatty acids (e.g., arachidonic acid) augment this process. Ferroptosis inhibition reduces sEVs release and increases ERS sensitivity, revealing its cytoprotective role (90).

HMGB1/RICTOR upregulate PD-L1 expression and PD-L1+ sEVs release to impair immune function and anti-PD-L1 efficacy. They also enhance glutaminolysis via mTORC2-AKT-c-MYC (upregulating GS) and mTORC1-mediated GDH derepression (91) (**Table 2**).

**Table 2:** Main mechanisms involved in the regulation of metabolic networks by sEVs.

Metabolic type	Key signals	Main mechanisms	Ref.
Lacking oxygen	lncRNA HMMR-AS1	Activates HIF-1α and significantly increases lncRNA HMMR-AS1 expression. Delivery to macrophages via sEVs induces M2-type polarization	(138)
	Glycosaminoglycan-3	Activation of HIF-1α, reduction of GPC3 expression, inhibition of HCC cell proliferation, migration and epithelial-mesenchymal transition, inhibition of Wnt/β-catenin signaling pathway, inhibition of tumor growth and angiogenesis	(76)
	miR-3174	Activation of HIF-1α, up-regulation of miR-3174 expression, delivery to human umbilical vein endothelial cells via sEVs, inhibition of HIPK3 signaling pathway, enhancement of angiogenesis and vascular permeability, and promotion of HCC growth and metastasis	(77)
	miR-1273f	Activation of Wnt/β-catenin signaling pathway enhances proliferation, migration, invasion, and epithelial-mesenchymal transition of HCC cells	(78)
	oleanolic acid	Inhibition of ERK1/2-NFκB signaling pathway and effective prevention of hypoxia-induced formation of distal pre-metastatic microenvironment	(81)
	CircHIF1A	Binds to HuR, stabilizes PD-L1 expression, and enhances proliferation, migration, invasion and epithelial-mesenchymal transition of HCC cells. Inhibits cytotoxicity of CD8+ T cells, leading to immune escape	(82)
Glycolysis	lncMMPA	Competes with miR-548s for binding, increases ALDH1A3 expression, and promotes glycolytic activity and proliferation of HCC cells	(139)
	lncRNA TUG1	Inhibition of miR-524-5p, up-regulation of SIX1 expression, and promotion of glycolysis-related gene activity	(140)
	ZFPM2-AS1	Inhibition of miR-18b-5p, enhancement of PKM expression, activation of the HIF-1α-dependent glycolytic pathway, promotion of proliferation, migration and invasion of HCC cells, and promotion of M2-type polarization in tumor-associated macrophages	(83)
	miR4458HG	Binds to the m6A reader IGF2BP2, stabilizes mRNAs of glycolysis-related genes, and enhances the glycolytic process in HCC cells	(84)
	CircFBLIM1	Reduction of LRP6 inhibition by miR-338, promotion of LRP6 expression, enhancement of glycolysis and tumor progression in HCC cells	(85)
	CircZNF652	Reduction of GUCD1 inhibition by miR-29a-3p, enhancement of glycolysis-related metabolic activities such as glucose uptake, pyruvate levels, lactate production and ATP production	(86)
	miRNA-146a-5p	IRF7 upregulates the expression of PFKL, a key enzyme in glycolysis. Targeted inhibition of IRF7 reduces glucose uptake, lactate production and ATP yield. Accelerates cellular senescence	(88)
M6a modification	HBeAg	Enhancement of m6A methylation modification and stabilization of MAAS in M2-type macrophages. delivery of MAAS to HBV-associated HCC cells via sEVs and promotion of tumor cell proliferation	(141)
	miR-628-5p	Delivery of miR-628-5p into HCC cells via sEVs, inhibition of METTL14 expression, reduction of m6A modification of circFUT8, and blocking of circFUT8 translocation from nucleus to cytoplasm	(142)
	FTO	Removal of m6A modification on GPNMB mRNA, stabilization of GPNMB expression, binding of GPNMB to SDC4 receptor on CD8+ T cells via sEVs, inhibition of T cell activation, promotion of immune escape	(89)
Other metabolism	HMGB1和RICTOR	Modulation of PD-L1 expression, promotion of PD-L1+ sEVs generation, inhibition of cytotoxicity in immune cells, and attenuation of the effects of anti-PD-L1 immunotherapy	(91)
	HMGB1和RICTOR	Activation of the mTORC2-AKT-C-MYC pathway, upregulation of glutamine synthetase expression, deregulation of glutamate dehydrogenase inhibition, and enhancement of glutamine metabolism	(91)

sEVs bilayers contain phosphatidylserine, sphingomyelin, and cholesterol, with LPC modulating membrane stability/function. Exogenous LPC/PGD2 activate TGF-β via TLR2/DP1 to promote fibrosis/immunomodulation. Cholesterol-conjugated siRNAs enhance sEVs delivery efficiency, while vitamin E modifications improve cargo loading (92–94). miR-23b-3p is enriched in sEVs from aged mice/FH patients, accelerating senescence/metabolic dysfunction via Tnfaip3 suppression. Targeting miR-23b-3p may treat age-related liver/metabolic disorders (95). While sEVs roles in HCC lipid metabolism require further study, their regulatory functions show significant research value.

### 3.3 sEVs in immune network regulation

#### 3.3.1 sEVs and macrophages

Macrophage-derived sEVs play crucial regulatory roles in HCC invasive phenotypes. Studies demonstrate macrophages enhance HCC invasiveness by secreting miR-92a-2-5p-enriched sEVs. These sEVs are internalized by HCC cells to downregulate androgen receptor (AR) expression, activating the AR/PHLPP/p-AKT/β-catenin signaling axis and promoting tumor progression. Experimental evidence shows inhibiting sEVs secretion or miR-92a-2-5p knockdown significantly attenuates macrophage-mediated HCC invasion (96).

sEVs-mediated immune evasion and immunosuppression in HCC microenvironment have been extensively investigated. HCC-derived sEVs deliver PCED1B-AS1 to T cells/macrophages, reducing hsa-miR-194-5p to upregulate PD-L1/PD-L2, inducing immune cell apoptosis/dysfunction (97). GOLM1 facilitates PD-L1 transfer via HCC-sEVs to tumor-associated macrophages (TAMs), enhancing immune evasion and CD8+ T cell suppression. Zoledronic acid combined with anti-PD-L1 effectively reverses this immunosuppression (98).

Recent breakthroughs reveal therapeutic potential of macrophage-derived sEVs in HCC. RBPJ-overexpressing macrophage sEVs (RBPJ+/+ Mφ-Exo) deliver hsa_circ_0004658 to suppress HCC proliferation and induce apoptosis. This circRNA sponges miR-499b-5p to derepress JAM3, exerting antitumor effects (99). Conversely, HCC-derived circTMEM181-enriched sEVs upregulate macrophage CD39, activating ATP-adenosine pathway to create immunosuppressive microenvironment and impair anti-PD1 efficacy (100). These findings provide novel directions for sEVs-targeted HCC therapies.

#### 3.3.2 sEVs and M1 macrophages

HBV-associated HCC sEVs exhibit significant miR-142-3p upregulation. These sEVs deliver miR-142-3p to induce M1 macrophage ferroptosis, promoting tumor progression. Mechanistically, miR-142-3p targets SLC3A2 to regulate macrophage ferroptosis. This reveals how sEVs promote HBV+ HCC by modulating macrophage function (101). Additionally, HCC-derived sEVs can drive M1 macrophage polarization. FTCD-mediated sEVs signaling promotes M1 polarization to suppress HCC proliferation (102).

sEVs combined with superparamagnetic iron oxide nanoparticles (PIONs@E6) enhance M1 polarization. This combination increases proinflammatory cytokines (IL-12, TNF-α) and ROS production, effectively suppressing HCC growth in mice. sEVs-nanoparticle conjugates enhance macrophage antitumor immunity (103). A novel sEVs-mimetic nanosystem reprograms immunosuppressive M2 TAMs to antitumor M1 phenotype. Near-infrared irradiation increases M1 macrophages, inhibits tumor growth, and enhances immune activity in TME (104).

#### 3.3.3 sEVs and M2 macrophages

HCC cells regulate macrophage polarization via sEVs secretion to promote tumor development. HCC-sEVs deliver hsa_circ_0074854 and other ncRNAs to induce M2 polarization, enhancing migration/invasion. hsa_circ_0074854 inhibition reverses this effect, confirming its key role in tumor-immune crosstalk (105).

sEVs-delivered miRNAs promote M2 polarization by targeting specific genes. miR-452-5p and miR-21-5p downregulate TIMP3 and RhoB respectively, enhancing HCC malignancy (106, 107). miR-200b-3p reinforces M2 polarization via ZEB1/JAK/STAT pathway (108). IL-6-stimulated HCC cells secrete miR-143-3p-enriched sEVs that promote M2 polarization via MARCKS regulation (109).

M2 macrophage-derived sEVs reciprocally promote tumor progression. Their miR-27a-3p and miR-660-5p suppress TXNIP and KLF3 respectively, enhancing HCC stemness/invasiveness (110, 111). lncRNAs (PSMA5, HEIH) in HCC-sEVs activate JAK2/STAT3 to induce M2 polarization (112, 113).

M2 macrophage sEVs mediate HCC drug resistance and vascular remodeling via specific miRNAs. miR-200c-3p activates PI3K/AKT pathway to induce sorafenib resistance (114). miR-23a-3p targets PTEN/TJP1 to disrupt vascular barriers and promote metastasis (115). These findings reveal multifaceted regulatory roles of sEVs in HCC microenvironment.

#### 3.3.4 sEVs and T lymphocytes

HCC-derived sEVs modulate immune cell functions within the tumor microenvironment through multiple mechanisms, thereby influencing tumor progression. HCC-derived sEVs deliver 14-3-3ζ protein to tumor-infiltrating lymphocytes (TILs), impairing their activation, proliferation, and antitumor functions while accelerating T cell exhaustion. This mechanism demonstrates how sEVs suppress TIL immunocompetence to attenuate antitumor responses and promote HCC progression (116). Furthermore, sEVs play pivotal roles in regulatory T cell (Treg) expansion. HCC-sEVs carrying circGSE1 activate the TGFBR1/Smad3 pathway by sponging miR-324-5p, thereby enhancing Treg-mediated immunosuppression. This process inhibits CD8+ T cell antitumor activity and facilitates HCC immune evasion (117).

Multiple studies have investigated HCC-sEVs regulation of dendritic cells (DCs) and DC-mediated T cell responses. For instance, HCC-sEVs are internalized by DCs to present tumor antigens and activate CD8+ T cells, inducing antitumor immunity. However, sEVs concurrently suppress DC IL-12 secretion, which can be restored by IL-12 supplementation to enhance CTL-mediated tumor killing (118, 119).

The immunomodulatory properties of sEVs confer potential as antitumor vaccines. DC-derived sEVs (Dex) combined with microwave ablation (MWA) enhance CD8+ T cell infiltration while reducing Tregs, remodeling the immunosuppressive microenvironment comparably to DC vaccines (120). Moreover, tumor antigen-loaded sEVs potently enhance T cell function, demonstrating robust antitumor activity both *in vitro* and in murine models (121, 122).

The synergy between sEVs and immune checkpoint inhibitors has garnered significant attention. Antigen-loaded DC-derived sEVs (DC-TEX) increase intratumoral CD8+ T cells and elevate IFN-γ/IL-2 cytokine levels. Combined with anti-PD-1, they reverse T cell exhaustion and significantly enhance antitumor immunity (123). Beyond antitumor immunity, sEVs exhibit potential in antiviral immunity. HDV antigen-loaded DC-sEVs activate CD8+ T cells and promote Th1 responses via JAK/STAT signaling to suppress HDV replication (124).

#### 3.3.5 sEVs and natural killer cells

HCC-derived sEVs significantly regulate natural killer (NK) cell immune functions. HCC-sEVs deliver miR-92b to NK cells, downregulating CD69 expression and impairing cytotoxicity to facilitate immune evasion (125). Additionally, HCC-sEVs transfer circUHRF1 to downregulate miR-449c-5p and upregulate TIM-3, inducing NK cell exhaustion and impairing anti-PD1 efficacy (126). Another mechanism involves miR-17-5p transfer, which suppresses the RUNX1-NKG2D axis to further compromise NK cell tumoricidal activity (127).

In contrast, NK cell-derived sEVs (NK-exo) enriched with cytotoxic proteins induce HCC apoptosis by inhibiting AKT/ERK1/2 signaling (73). IL-15/IL-21-stimulated NK-exos exhibit enhanced antitumor activity due to elevated cytotoxic protein content (128).

HCC-mediated immunosuppression via sEVs reveals novel immune escape mechanisms, while NK-exos demonstrate therapeutic potential. Future studies should explore blocking protumor sEVs or leveraging NK-exos to enhance antitumor immunity.

#### 3.3.6 sEVs and fibroblasts

Cancer-associated fibroblast (CAF)-derived sEVs regulate HCC migration/invasion via noncoding RNAs. Reduced miR-150-3p in CAF-sEVs enhances HCC migratory/invasive capacities. Low miR-150-3p correlates with poor HCC prognosis, suggesting its regulatory role (129). CAF-sEVs deliver miR-92a-3p to activate Wnt/β-catenin signaling, promoting HCC proliferation/stemness (130). CAFs also modulate tumor suppressors to influence HCC progression. CAF-sEVs transfer miR-20a-5p to suppress LIMA1 and enhance HCC malignancy (131).

During metastasis, B[a]P-treated HCC cells transfer circ_0011496 via sEVs to activate lung fibroblasts into CAFs. This circRNA enhances profibrotic/proinflammatory functions via miR-486-5p/TWF1/MMP9 to drive pulmonary metastasis (132). Conversely, CAF-sEVs-delivered miR-29b suppresses metastasis by downregulating DNMT3b and upregulating MTSS1 (133).

Regarding chemoresistance, CAF-sEVs-circZFR enhances cisplatin resistance by inhibiting STAT3/NF-κB signaling (134). sEVs-transferred Gremlin-1 reduces sorafenib sensitivity via EMT and Wnt/β-catenin/BMP pathway modulation (135). These findings highlight CAF roles in HCC TME and suggest therapeutic strategies (**Table 3**).

**Table 3:** Main mechanisms involved in the regulation of immune networks by sEVs.

Immune cell	Key signals	Main mechanisms	Ref.
Macrophage	miR-92a-2-5p	Reduction of androgen receptor expression, enhancement of hepatocellular carcinoma cell invasiveness, and modulation of AR/PHLPP/p-AKT/β-catenin signaling pathway	(96)
	PCED1B-AS1	Release of PCED1B-AS1 via sEVs, resulting in decreased levels of hsa-miR-194-5p in immune cells. Increases PD-L1 and PD-L2 expression, triggering apoptosis and decreased viability of immune cells.	(97)
	GOLM1	Promotion of PD-L1 stability, delivery of PD-L1 to tumor-associated macrophages via sEVs, increase of PD-L1 expression on macrophages, enhancement of immune escape, inhibition of CD8+ T cell activity	(98)
	RBPJ	Carrying up-regulated hsa_circ_0004658 by sEVs, competitive adsorption of miR-499b-5p, deregulation of JAM3, and up-regulation of JAM3 expression	(99)
	CircTMEM181	Delivery of circTMEM181 to macrophages via sEVs, promotion of CD39 expression in macrophages, activation of the ATP-adenosine pathway, formation of an immunosuppressive microenvironment, and weakening of the antitumor effect of CD8+ T cells	(100)
	lncMMPA	Interacts with miR-548s, enhances ALDH1A3 expression	(139)
M1 macrophage	miR-142-3p	Delivery of miR-142-3p via sEVs, targeted down-regulation of SLC3A2 expression, and induction of iron death in M1-type macrophages	(101)
	FTCD	Promotion of macrophage polarization to M1 type by sEVs	(102)
	miR-628-5p	Delivery of miR-628-5p into HCC cells via M1-Exo, inhibition of METTL14 expression, reduction of m6A modification of circFUT8, and blocking of circFUT8 translocation from nucleus to cytoplasm	(142)
	PIONs@E6	Significantly promotes polarization of M1 macrophages	(103)
	MPDA/ICG@M1NVs	Repolarization of immunosuppressive M2 tumor-associated macrophages into anti-tumor M1 macrophages	(104)
M2 macrophage	Hsa_circ_0074854	Delivery of hsa_circ_0074854 to macrophages and promotion of macrophage M2 polarization via sEVs	(105)
	miR-452-5p	Targeting TIMP3 and promoting M2 polarization in macrophages	(106)
	miR-21-5p	Targeting RhoB and promoting M2 polarization in macrophages	(107)
	miR-200b-3p	Inhibition of ZEB1, activation of the JAK/STAT signaling pathway, and promotion of M2 polarization in macrophages	(108)
	miR-143-3p	Regulation of the MARCKS gene in TAMs, promotion of M2 polarization in macrophages	(109)
	miR-27a-3p	Down-regulation of TXNIP gene, enhancement of stemness characteristics and malignant behavior of HCC cells	(110)
	miR-660-5p	Down-regulation of KLF3 gene, enhancement of stemness characteristics and malignant behavior in HCC cells	(111)
	PSMA5	Activation of JAK2/STAT3 pathway, induction of M2 polarization in macrophages	(112)
	HEIH	Activation of miR-98-5p/STAT3 pathway, induction of M2 polarization in macrophages	(113)
	HMMR-AS1	Delivery to macrophages via sEVs, competitive adsorption of miR-147a, prevention of its degradation by ARID3A, promotion of M2 polarization	(138)
	LncRNA MAPKAPK5_AS1	Delivery of MAAS via sEVs and promotion of HCC cell proliferation. HBeAg stabilizes MAAS expression in M2 macrophages by enhancing m6A methylation modification	(141)
	miR-200c-3p	Activation of PI3K/AKT signaling pathway and enhancement of sorafenib resistance in HCC cells	(114)
	miR-23a-3p	Targeting PTEN and TJP1, increasing vascular permeability, and weakening intercellular tight junctions	(115)
T lymphocyte	14-3-3ζ protein	Delivery of 14-3-3ζ protein to tumor-infiltrating T lymphocytes via sEVs results in decreased T cell activation and proliferation, reduced anti-tumor activity, and a greater tendency to depletion	(116)
	CircGSE1	Acts as a sponge for miR-324-5p, activates the TGFBR1/Smad3 signaling pathway, enhances the function of Tregs, and inhibits the anti-tumor activity of effector T cells	(117)
	Rab27a	Increased secretion of sEVs, significant promotion of dendritic cell maturation, expression of higher levels of MHC class II molecules and co-stimulatory molecules CD80 and CD86, inhibition of IL-12 secretion by DCs	(119)
Natural killer cell	miR-92b	Delivery of miR-92b to natural killer cells via sEVs, inhibition of CD69 expression, and impaired cytotoxicity of NK cells	(125)
	CircUHRF1	Degradation of miR-449c-5p and up-regulation of TIM-3 expression, inhibition of anti-tumor activity of NK cells	(126)
	miR-17-5p	Delivery of miR-17–5 to natural killer cells via sEVs, inhibition of RUNX1-NKG2D axis expression, and attenuation of cytotoxicity and killing capacity of natural killer cells	(127)
Fibroblast	miR-150-3p	Reduction of miR-150-3p significantly enhances migration and invasion of HCC cells	(129)
	miR-29b	Down-regulation of DNA methyltransferase 3b and up-regulation of tumor suppressor MTSS1	(133)
	Circ_0011496	Activation of lung fibroblasts, promotion of their transformation to CAFs, regulation of the miR-486-5p/TWF1/MMP9 axis, and enhancement of pro-fibrotic and pro-inflammatory functions of fibroblasts	(132)
	miR-92a-3p	Inhibition of AXIN1 gene expression, activation of the Wnt/β-catenin signaling pathway, significant promotion of proliferation and stemness characteristics of HCC cells, and enhancement of tumor invasiveness and metastatic potential	(130)
	CircZFR	Enhancement of cisplatin resistance and inhibition of STAT3/NF-κB signaling pathway in HCC cells	(134)
	lncRNA TUG1	Inhibition of miR-524-5p and up-regulation of SIX1 gene expression	(140)
	Gremlin-1 protein	Enhancement of epithelial-mesenchymal transition, modulation of Wnt/β-catenin and BMP signaling pathways, and reduction of sensitivity to sorafenib in HCC cells	(135)
	miR-20a-5p	Inhibition of the expression of the oncogene LIMA1	(131)

#### 3.3.7 sEVs and complement system

sEVs employ complement regulators for self-protection. Surface CD55/CD59 inhibit membrane attack complex (MAC) formation to prevent complement-mediated lysis. This enhances sEVs stability in bodily fluids for prolonged immunomodulation. Antigen-presenting cell-derived sEVs maintain structural integrity via this mechanism (136).

sEVs modulate complement via C3 fragments. B cell/macrophage-derived sEVs containing C3 fragments promote complement activation. This enhances antigen presentation and T cell responses. C3 fragments may also confer additional complement resistance (137).

### 3.4 sEVs in multi-network regulation

**Figure 3:**
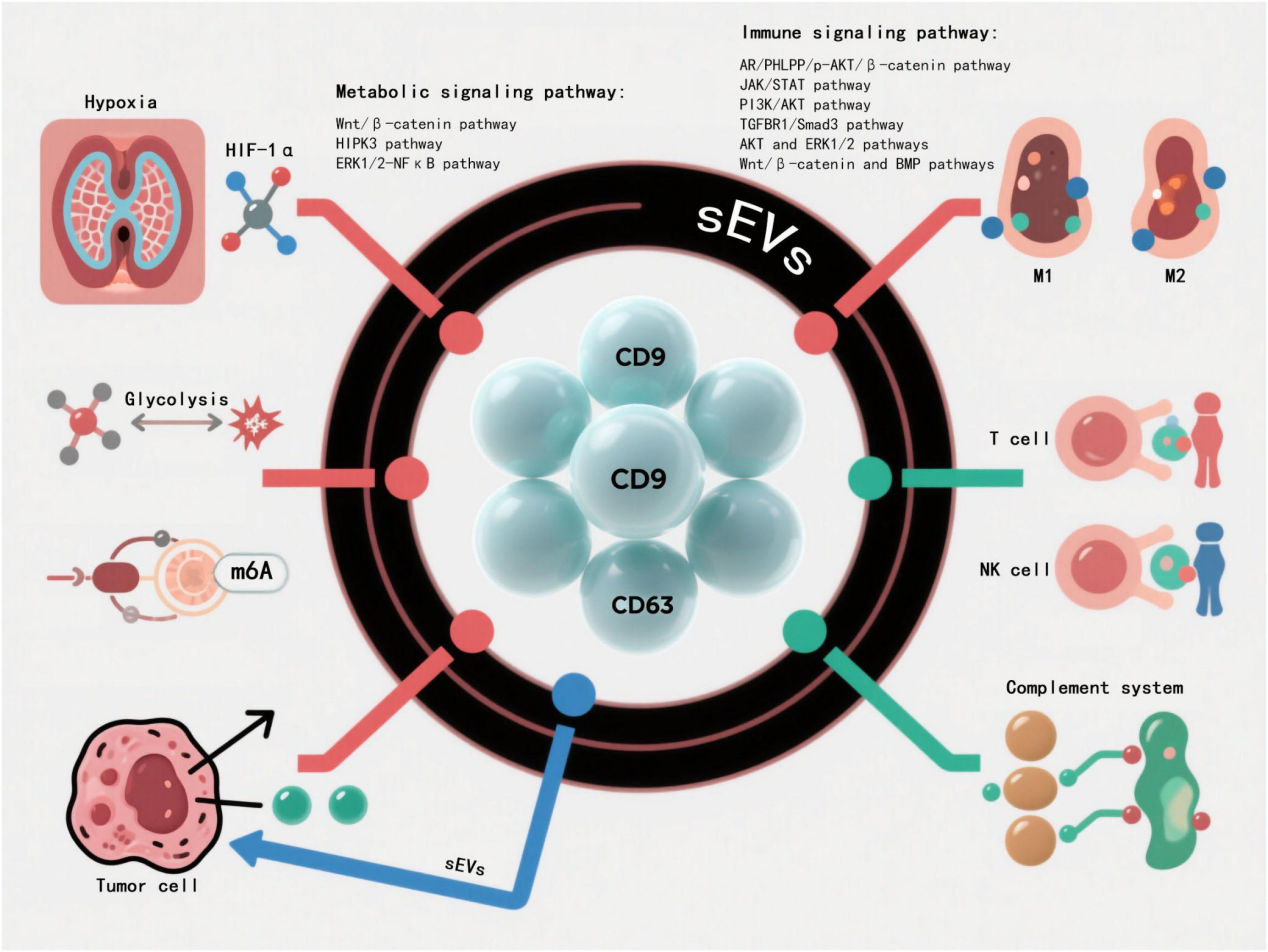
sEVs play a core role in multi-network regulation. sEVs systematically integrate into the highly complex intercellular communication network system through the bioactive molecular libraries such as proteomics,nucleic acid components and lipid groups they carry. The metabolic-immune cross-regulatory network mediated by them shows multi-dimensional regulatory characteristics, among which multiple signal transduction pathways constitute the key molecular hubs of cascade regulation.

sEVs participate in complex intercellular communication networks by transporting bioactive molecules including proteins, nucleic acids, and lipids. They play pivotal roles in metabolic regulation and immunomodulation: modulating insulin sensitivity, glycolipid metabolic enzyme activity and mitochondrial function to maintain energy homeostasis, while precisely controlling immune responses through antigen presentation, immune receptor interactions and cytokine regulation. This regulation exhibits high specificity depending on sEVs cargo composition and microenvironmental conditions (**Figure 3**).

In HCC, sEVs-mediated intercellular communication significantly influences tumor progression. Hypoxic conditions activate HIF-1α, promoting HCC cells to secrete HMMR-AS1 lncRNA-enriched sEVs. Upon macrophage uptake, these vesicles competitively bind miR-147a to upregulate ARID3A, inducing M2 polarization that enhances immunosuppression and accelerates tumor progression (138).

Tumor-associated macrophage (TAM)- and cancer-associated fibroblast (CAF)-derived sEVs regulate HCC metabolism through noncoding RNA delivery. TAM-secreted lncMMPA suppresses miR-548s to upregulate ALDH1A3, enhancing glycolysis and tumor proliferation (139). Similarly, CAF-derived TUG1 inhibits miR-524-5p to activate SIX1, promoting glycolysis and invasive capacity (140). These findings demonstrate the central role of sEVs in HCC metabolic reprogramming.

In HBV-associated HCC, HBeAg stabilizes lncRNA MAAS in macrophages via m6A modification, promoting its enrichment in sEVs. MAAS delivery to HCC cells significantly enhances proliferation (141). Conversely, M1 macrophage-derived sEVs deliver miR-628-5p to suppress METTL14-mediated m6A modification of circFUT8, thereby inhibiting tumor growth (142). This contrast highlights the bidirectional regulation of HCC by sEVs through epigenetic mechanisms.

## 4 Therapeutic applications of sEVs in hepatocellular carcinoma microenvironment

sEVs play a central role in regulating the HCC microenvironment. By mediating the transfer of various oncogenic molecules and signaling pathways, sEVs critically regulate the formation and evolution of the HCC tumor microenvironment. In HCV-associated HCC, CD81+ sEVs significantly impair host immune surveillance through immune evasion mechanisms, establishing them as promising therapeutic targets (59). The Rab27B-dependent sEVs-mediated drug efflux mechanism has been shown to substantially enhance chemoresistance in HCC cells (51). HBV core antigen (HBc) upregulates miR-135a-5p in sEVs to inhibit VAMP2 function, promoting anti-apoptotic properties and drug resistance in HCC cells (60). These studies elucidate the molecular mechanisms of sEVs-mediated therapy resistance in HCC and identify multiple potential targets for therapeutic intervention.

sEVs exhibit multifaceted regulatory functions in HCC immune evasion. sEVs surface-associated immune checkpoint molecules like PD-L1 effectively suppress T cell antitumor activity, reducing clinical response to immunotherapy (82, 91, 97, 98). Under hypoxic conditions, sEVs selectively enrich and deliver specific miRNAs/lncRNAs to enhance immunosuppression and accelerate HCC progression (76–78, 81, 82, 138).

With inherent biocompatibility and targeting capabilities, sEVs offer distinct advantages for drug delivery systems. Nanoengineered sEVs significantly improve targeting precision and bioavailability of therapeutic agents (68, 103, 104, 143). For gene/immunotherapies, sEVs demonstrate remarkable clinical potential by efficiently delivering functional nucleic acids or immunomodulators to enhance T cell activation and antitumor immunity (118, 122).

Artificial intelligence is transforming methodological approaches in sEVs research. Machine learning algorithms significantly enhance TEM and cryo-EM capabilities for sEVs ultrastructural analysis, enabling automated classification and quantification. AI-driven multi-omics integration efficiently identifies sEVs-associated diagnostic biomarkers, with random forest models demonstrating reliability for liquid biopsy applications. Computational biology frameworks integrate sEVs-mediated intercellular networks with tumor ecosystem dynamics, providing novel paradigms for studying oncogenesis.

## 5 Conclusion

HCC ranks among the most prevalent and lethal malignancies worldwide. Emerging fundamental research demonstrates that sEVs play pivotal regulatory roles in HCC pathogenesis and progression. As crucial intercellular communication vehicles, sEVs orchestrate HCC initiation, progression and malignant transformation through complex molecular networks encompassing cellular communication, metabolic regulation and immunomodulation.

By transporting diverse bioactive molecules (miRNAs, proteins, lipids), sEVs establish sophisticated signaling networks between cancer cells and microenvironmental components. These molecules enhance cancer cell proliferation, invasion and metastatic potential. Specifically, sEVs-enclosed miRNAs can selectively suppress tumor suppressor genes to accelerate HCC malignancy. Furthermore, sEVs reinforce malignant phenotypes by reprogramming cancer cell gene expression profiles.

Regarding metabolic regulation, sEVs modulate HCC metabolic characteristics through multiple mechanisms. They transfer critical metabolic enzymes/regulators and substantially alter glucose, lipid and energy metabolism pathways in HCC cells. Studies show sEVs enhance aerobic glycolysis via specific metabolic enzymes, sustaining proliferative capacity even under hypoxic conditions.

In immunomodulation, sEVs critically contribute to HCC immune evasion. They deliver immune checkpoint molecules (e.g., PD-L1) to impair immune surveillance and tumor clearance. Concurrently, sEVs modulate tumor-associated macrophage polarization while suppressing T/NK cell antitumor activity, establishing an immunosuppressive niche favorable for tumor growth.

In summary, sEVs comprehensively participate in shaping the HCC microenvironment by integrating cellular communication, metabolic reprogramming and immune evasion networks. These findings not only deepen our understanding of HCC pathogenesis but also provide theoretical foundations for novel diagnostic/therapeutic strategies. Future studies should further elucidate sEVs molecular mechanisms in HCC and explore clinical applications of sEVs-based targeted therapies and drug delivery systems. With advancing research, sEVs may emerge as crucial breakthroughs in HCC diagnosis and treatment.
